# A Tumor Accelerator Based on Multicomponent Bone Scaffolds and Cancer Cell Homing

**DOI:** 10.3390/polym14163340

**Published:** 2022-08-16

**Authors:** Chen-Ji Huang, Pei-Kuan Chou, Zong-Yi Sher, You-Rong Chen, Tan-Yueh Chen, Guo-Chung Dong

**Affiliations:** 1 Institute of Biomedical Engineering and Nanomedicine, National Health Research Institutes, Miaoli 35053, Taiwan; 2 Graduate Program of Biotechnology in Medicine, National Tsing Hua University, Hsinchu 300044, Taiwan; 3 International Master Program of Translational Medicine, National United University, Miaoli 360301, Taiwan

**Keywords:** epidermal growth factor (EGF), MDA-MB-231, gelatin hydroxyapatite glutaraldehyde (GHG) scaffolds, microenvironment

## Abstract

Bone tissue attracts cancer cell homing biologically, mechanically, or chemically. It is difficult and time consuming to identify their complex cross-talk using existed methods. In this study, a multi-component bone matrix was fabricated using gelatin, hydroxyapatite (HAp), and epidermal growth factor (EGF) as raw materials to investigate how “acellular” bone matrix affects cancer cell homing in bone. Then, EGF-responsive cancer cells were cultured with the scaffold in a dynamical bioreactor. For different culture periods, the effects of HAp, gelatin, and EGF on the cell adhesion, proliferation, 3D growth, and migration of cancer were evaluated. The results indicated that a small amount of calcium ion released from the scaffolds accelerated cancer MDA-MB-231 adhesion on the surface of inner pores. Moreover, degradable gelatin key caused cancer cell growth on the scaffold surface to turn into a 3D aggregation. Despite this, the formation of cancer spheroids was slow, and required 14 days of dynamic culture. Thankfully, EGF promoted cancer cell adhesion, proliferation, and migration, and cancer spheroids were observed only after 3-day culture. We concluded that the combination of the multiple components in this scaffold allows cancer cells to meet multiple requirements of cancer dynamic progression.

## 1. Introduction

Cancer is a disease with a dynamic pattern that is unique to each individual. A developing tumor engages in continuous and complex multidimensional interactions with its surroundings, making it highly heterogeneous, while making prediction of its concomitant progression and treatment response more difficult. In recent years, tumor dynamics has emerged as a novel model for exploring specific elements of the tumor microenvironment that can be applied to predict tumor trajectories. The most basic component of tumor kinetics is to explore the molecular characteristics of tumors, including the study of tumor creation and the dimensional environment. This leads to a clearer understanding of how cancer develops, progresses, and metastasizes in response to treatment, leading to new therapies. Precise prevention, treatment, and care strategies can be provided as personalized medicine [1].

Tissue engineering has been used to simulate the tumor microenvironment in order to explore specific factors and to study related disease mechanisms and processes. This technique has been used in the construction of 3D cancer cultured models in order to investigate disease patterns. In particular, the focus has been on tumor stroma establishment and corresponding research related to individualized drug testing and precision medicine [2]. The components of three-dimensional culture scaffolds can mainly be divided into natural materials and synthetic materials, where natural materials include collagen, gelatin, chitosan, etc. [3]. Natural materials can also be combined with synthetic materials to produce composite scaffolds, such as the PCL–hydroxyapatite (HAp) scaffold [4]. At present, three methods—hydrogel, bioprinting, and microfluidics—are commonly used in three-dimensional cell cultures [5]. Among them, hydrogel-binding protein-based biomaterial inks have attracted attention due to their simulated properties (such as simulating extracellular matrix properties, etc.) [6]. There are, however, some shortcomings in the above methods, such as the lack of ability to adjust the biodegradability, stiffness, and morphology of hydrogels [7], the lower cell viability and adhesion of bioprinting, and less smaller amount of extracellular matrix produced by microfluidics [5]. Studies have shown that due to the multiple properties of hydroxyapatite, such as mechanical properties and antibacterial properties, it can be practically applied in the field of biomedicine [8]. In addition, studies have indicated that rigid scaffolds, such as gelatin–hydroxyapatite–glutaraldehyde (GHG) scaffolds [9], possess better cell adhesion, proliferation and migration capabilities [7,9,10,11]. In addition, it has been reported that immobilized EGF contributes to cell signaling, proliferation and migration functions [12,13,14]. However, the cellular microenvironment formed by the scaffold matrix combined with EGF has not been clearly described.

Nearly 10 million people died from cancer in 2020, with lung cancer and breast cancer being the leading causes of death [15]. Breast cancer is difficult to diagnose without obvious symptoms in the early stages, and bone pain symptoms often appear after cancer cells have metastasized to the bone [16,17,18]. If it is judged to be advanced cancer, the survival rate is only about 20 to 30% [19]. Even if the metastatic tumor is surgically removed, there may be residual cancer cells or some cancer cells that spread into the blood vessels during the removal process, leading to the risk of recurrence [20]. Therefore, it is necessary to understand breast cancer cell metastasis. In addition, breast cancer cells possess overexpressed epidermal growth factor receptor (EGFR) on the membrane surface [21,22], which is involved in various processes promoting tumorigenesis and metastasis [23]. In the bone metastasis stage, cancer cells release PTHrP to promote osteoblasts for the secretion of RANKL, and then RANKL further stimulates osteoblast activation and erodes bone tissue, thus producing a large amount of EGF and calcium ions. Furthermore, the malignant growth and proliferation of cancer cells is accelerated by these growth factors [24,25].

**Figure 1:**
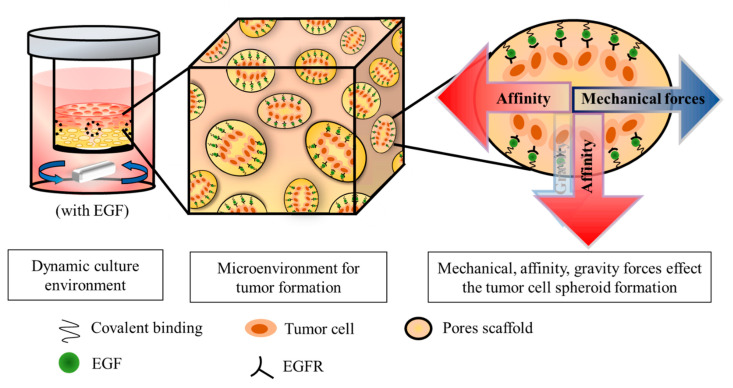
Schematic diagram of dynamic culture of tumor cells on GHG scaffold including immobilized EGF; in this way, the generated bio-affinity overcomes gravity and mechanical force to form a spheroid.

Both gravity from the cells themselves and centrifugal force (mechanical force) caused by stirring the mixed medium are present in the dynamic cell culture system. Therefore, the cell distribution is expected to be concentrated (adhesion and proliferation) in the upper portion of the scaffold. If EGF is immobilized on the scaffold, it is expected that the distribution of cells will be more widely distributed in the lower portion of the scaffold, since the strong biological affinity of EGF will counteract the above-mentioned mechanical force and dominate the cellular behaviors (as shown in Figure 1). Therefore, with the assistance of the affinity manifested by EGF, the cells adhering to the scaffolds not only have the ability to proliferate, but it is also implied that they have the ability to migrate.

In this study, we would like to illustrate the dynamic progression of cancer cells in bone metastasis by means of a multi-component scaffold matrix. The setting is based on the example of the metastasis of triple-negative breast cancer (MDA-MB-231) to humanoid bone scaffold. Firstly, the preparation of the bone-like scaffold will be described and the basic characteristics analyzed, and then the effects of the main matrices of the scaffold on the cellular behavior in dynamic culture, such as adhesion, proliferation, and migration, will be studied. At the same time, the relationship between our dynamic culture model and the hypoxia-driven cancer progression investigation are discussed. Finally, hypotheses about how the scaffold guides cell three-dimensional growth will be shown.

## 2. Materials and Methods

### 2.1. Cell Culture

Two human breast cancer cell lines, MDA-MB-231 (BCRC 60425) and MCF-7 (BCRC 60436), were obtained from the Bio-Resource Collection and Research Center (Hsinchu, Taiwan). Both cell lines were cultured in Dulbecco’s Modified Eagle Medium (DMEM) (Biological Industries, Kibbutz Beit-Haemek, Israel), including 10% Fetal Bovine Serum (FBS) (Peak serum, Colorado, CO, USA), 1% penicillin (Kibbutz Beit-Haemek, Israel), and 5% CO_2_ at 37 °C. The cells were passaged averaged every 3 days at 80% confluency.

### 2.2. Gelatin Hydroxyapatite Glutaraldehyde (GHG) Scaffold Preparation

The preparation of GHG scaffolds was mainly based on the articles published by Dr. Dong with minor modifications [26]. Firstly, 12 mL of deionized water was heated at 50 °C for 5 min and then 0.8 g gelatin (linear formula: (formula: C_40_H_59_N_11_O_13_)_n_) powder (St. Louis, MO, USA) was added and mixed by stirring at 1000 × RPM for 10 min. Then, 0.9 g of HAp (formula: Ca_5_(PO_4_)_3_(OH)) (St. Louis, MO, USA) was applied twice in sequence and stirred for 5 min, before adding 0.8 g of NaCl (St. Louis, MO, USA) and stirring for 10 min while passing through an air pump (Bio-Rad, Hercules, CA, USA) to inject air in order to create holes of various sizes (0.35 mL/min). When well mixed, the mixture was poured into a mold (diameter 15 mm) and sealed with paraffin and placed at −80 °C for 24 h. The formed semi-finished product was quickly cut (12 × 5 mm) and then placed in 1% glutaraldehyde (Fluka, Saint Louis, MO, USA) for 15 h at 25 °C with gentle shaking and protected from light. After washing with deionized water for 5 min, repeated three times, 0.2 M glycine (St. Louis, MO, USA) was added to neutralize the glutaraldehyde reaction with gentle shaking at 25 °C for 15 h. The sample was washed with deionized water for 10 min, repeated three times (or until the liquid after washing was no longer yellow), and then immersed in deionized water at 25 °C for 15 h with gentle shaking. The above washing steps were repeated three times, and then the scaffold was stored at −80 °C for at least 15 h and freeze dried (Labconco, Kansas City, MO, USA). The characteristics of the scaffold were determined using ImageJ, HE stain and SEM (Hitachi TM-1000, Tokyo, Japan), respectively. In addition, the surface morphology and structure of scaffolds were monitored using the SEM-EDX (Hitachi S-4700 Type-I, Tokyo, Japan).

### 2.3. Calcium Release Assay in GHG Scaffold

GHG scaffolds were soaked in 12 mL PBS for different durations at 37 °C, and then the calcium ion in the solution was measured using the Calcium Assay Kit (Abcam, Cambridge, UK) as the basis for calcium ion released. The measurement principle was the Calcium oCPC Method [27] which the o-cresolphthalein used to chelate with free calcium ions in the solution for producing a blue-violet complex by a spectrophotometer at 575 nm. The calcium concentration was calculated by interpolation of standard reagents.

### 2.4. EGF Immobilization

The EGF immobilization was mainly followed by Dr. Tseng’s publishing with minor modifications [28]. The scaffold was immersed in a well-mixed solution of 8 mM 1-ethyl-3-(3-dimethylaminopropyl)carbodiimide (EDC) and 10 mM N-hydroxysuccinimide (NHS) (Cytiva, Björkgatan, Uppsala, Sweden) for 1 h at 25 °C, then washed three times with phosphate-buffered saline (PBS, pH 7.4) (UniRegion Bio-Tech, New Taipei City, Taiwan), before being soaked in 170 nM streptavidin (St. Louis, MO, USA) solution (3 mL volume) and incubated at 4 °C for 5 h. After three washes with PBS, 6.61 nM Biotin-EGF (Thermo Fisher, Eugene, OR, USA) was reacted with streptavidin on the scaffold for 15 h at 4 °C. Finally, the EGF-GHG scaffold was synthesized after washing three times with PBS to remove unreacted EGF.

### 2.5. Dynamic Culture of Tumor Cells on Scaffold

The scaffold was irradiated with UV light for 30 min on laminar flow (15 min on the upper and lower sides) [29]. About 100–200 µL was applied, corresponding to approximately 2 million cells on the center of the 3.5 cm dish, and then seeded on the scaffold by capping. As a result of the porous property of the scaffold, the cell fluids infiltrated into the scaffolds from the bottom to the top via capillary action, and the scaffold was allowed to stand at 37 °C for 6 h to allow the cells to adhere to the bracket hole surface.

Before dynamic culture, the bioreactor (Figure S1) was soaked in 75% alcohol for 15 h, and then irradiated with UV light for 15 min. While the sterilization procedure was being completed, the bioreactor was washed twice with 5 mL of PBS and then once with 5 mL of culture medium. During the dynamic culture, firstly, 12 mL of culture solution was added to the outer container of the bioreactor and stirred in place; then, the syringe containing the inverted scaffold was carefully placed into the outer container and rotated through the stirrer below the inner container (50 × RPM), causing the disturbance of the culture medium to achieve the effect of internal and external flow, thereby simulating the environment of biological fluid circulation. The culture medium began to yellow significantly; therefore, the culture medium was replaced every one to two days. A schematic diagram of the process of cells adhered on scaffolds and dynamic culture is provided in Figure S2.

### 2.6. MDA-MB-231 Proliferation Analysis

Cultured scaffolds (180π mm^3^, 0.0831 ± 0.02 g) were degraded with 2 mL of collagenase (St. Louis, MO, USA) solution (2000 µg/mL) and incubated for 40 min at 37 °C, and then the reaction was stopped by adding 5 mL of DMEM medium and centrifuging (1000 × RPM, 5 min) to receive the supernatants. To perform the cell count, 1 mL of supernatant was drawn.

### 2.7. Effects of Scaffold Components and EGF on Cell Growth

After analyzing the components of the synthetic scaffold (Section 2.2), the MDA-MB-231 was seeded and cultured dynamically for three days to observe the cell adhesion on the main components. The cultured scaffold was treated by washing three times with PBS, and then fixed with 10% formalin at 25 °C for 24 h. Subsequently, the scaffold was gradient dehydrated with 30%, 40%, 50%, 60%, 70%, 80%, and 90% alcohol for 30 min each in sequence. The next stage was soaking with dehydrated alcohol for 20 h and 30 min, respectively. The cultured anhydrous scaffold was subjected to a critical point drying process and subsequently gold coated in preparation for SEM.

In addition, the adhered cell growth was also investigated following the immobilization of EGF on the surface of the above two materials.

### 2.8. Effects of EGF on the Proliferation of MDA-MB-231 in Dynamic Culture

The seven-day dynamic cultured scaffold was used for proliferation analysis. Except for the SEM, which was performed as described in Section 2.7, the HE and Ki-67 were performed as described below. The cultured scaffold was washed three times with PBS and then fixed with 10% formalin at 25 °C for 24 h. The treated scaffold was soaked with 75% alcohol, before being embedded in paraffin and sliced into tissue sections with a thickness of about 4–8 µm for HE staining. For the Ki-67 assay, the rabbit anti-Ki-67 antibody solution (1:200) was used as the primary antibody (Bethyl, Montgomery, AL, USA) and then the anti-rabbit mouse antibody was added as the secondary antibody (Bethyl, Montgomery, AL, USA) and reacted with it. Finally, substrate was added for the protein colorimetric assay. The quantity and expression of the Ki-67 protein were observed by microscope.

### 2.9. Effects of EGF on the Growth and Distribution of MDA-MB-231 on Scaffold

The three-day dynamic cultured scaffold was taken out and placed in a 24-well dish and washed three times with PBS, followed by fixing with 10% formalin (Macron Fine Chemicals, Center Valley, PA, USA), and incubated for 24 h at 25 °C. Then, it was treated with 75% alcohol and embedded in paraffin, before being sectioned from top to bottom (0th, 1000 and 1400 μm) and stained with immunohistochemical staining. The cell distribution on the scaffold slices was observed with a microscope.

## 3. Results

### 3.1. Preparation of Gelatin Hydroxyapatite Glutaraldehyde (GHG) Scaffold

**Figure 2:**
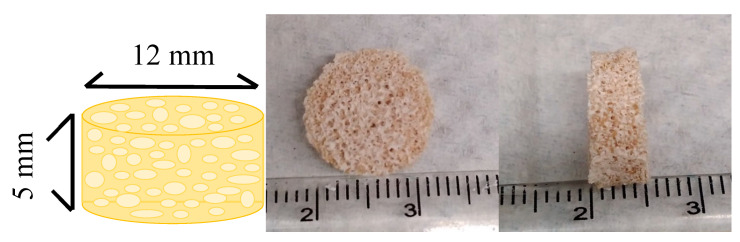
Analysis of the shape of the imitation bone scaffold: the scaffold was cylindrical, with a porous appearance, and a diameter and thickness of 12 and 5 mm, respectively.

Human bone is mainly composed of 70% inorganic and 30% organic material; therefore, HAp and gelatin were used as the raw materials for the imitation bone scaffold. GHG scaffolds with dimensions of were 12 × 5 mm (diameter × thickness) were prepared by cross-linking and freeze-drying, and consisted of beige-yellow porous structures of various sizes, demonstrating interconnected porous structures (Figure 2). The hole size and porosity calculated using Image J software were about 100 to 500 μm and 80%, respectively. This scaffold was similar to the porous structure of human bone trabeculae connected to each other in tissue sections, which could be used as an appropriate space in which cells could adhere and grow.

### 3.2. Analysis of Micro-Characteristics of GHG Scaffold

**Figure 3:**
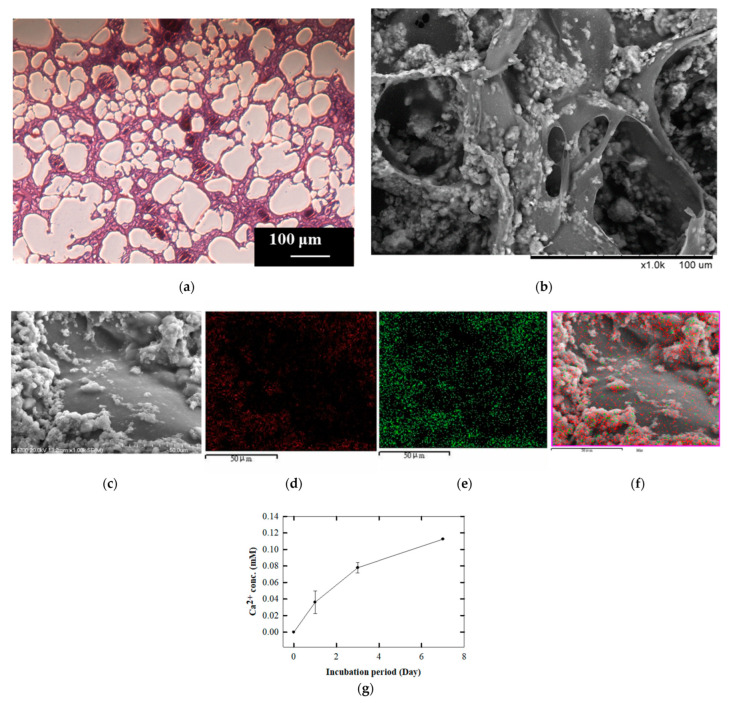
Analysis of the composition of the GHG scaffold: the protruding particle part in both the (**a**) HE stain and (**b**) SEM images indicate the HAp particles on the scaffold surface; the smooth part indicate the gelatin. (**c**) The SEM as changing the magnification showed the rough parts of the scaffold surface more clearly; EDX was used to analyze the distribution of (**d**) calcium ions and (**e**) phosphorus ions, respectively; merged results of (**c**–**e**) pointed out that large amount of calcium ions was observed in the rough portion, as shown in (**f**). (**g**) Calcium ions released from the scaffold and free calcium ion concentration increased with time. (Scale bar: 100 µm for (**a**,**b**); 50 µm for (**c**) to (**f**)). (The “×” in scale bar in the (**c**) represents magnification).

The configuration of the holes could be clearly observed with the naked eye after cutting the scaffold in half. To better understand the components inside the scaffold, we sliced the scaffold and stained it with HE, revealing an interconnected porous structure (Figure 3a). The pore size and porosity calculated using Image J were approximately 100 to 500 µm and 80%, respectively. These values are similar to the interconnected porosity of human trabecular bone in tissue sections [30]. These cavities provide an appropriate space in which cells can adhere and grow. Subsequently, it was observed from the SEM results that rough particles of different sizes were present on the surface of the scaffold. The literature reports that the content of rough particles mostly comprised calcium and phosphorus [31,32,33], from which it can be inferred that this region of rough particles was HAp (protruding particle part in Figure 3b). During the preliminary scaffold synthesis, the gelatin was in a smooth gelatinous state at 50 °C; therefore, it was speculated that the smooth area was composed of gelatin (smooth part in Figure 3b). The dark brown particles (a small number) and the light purple particles (mostly) were identified on the basis of HE staining as HAp and gelatin, respectively (Figure 3a).

In order to verify the composition of the rough part of the scaffold surface, we analyzed with different magnifications of SEM and EDX, respectively. The results showed that a clearer rough part was observed after magnification (Figure 3c); after merging the analysis result of distribution of calcium ions and phosphorus ions by EDX (Figure 3d,e) with the SEM result, a large amount of calcium ions distribution in the rough part were clearly observed (Figure 3f). Due to incompatibility with gelatin and causing heterogeneous aggregation and distribution during the preparation of the scaffold, we combined with the phenomenon of a large amount of calcium ion distribution in the rough part, it was presumed that the rough part observed on the surface of the scaffold was presumed to be HAp.

The calcium OCPC method [27] was used to measure free calcium ions. The results showed that, under the simulated physiological state, calcium ions were released from the scaffold into the free state along with increasing incubation time (Table 1, Figure 3c). Following the release of calcium ions from the scaffold, the observation of its structural changes on the basis of H&E tissue section indicated that the pores of the scaffolds exhibited connectivity following immersion for 7 days. This is consistent with the special properties of porosity and connectivity between pores displayed by human bone slices [31]. Therefore, it was confirmed that the prepared GHG scaffold was similar in structure to the human trabecular bone structure (Figure 3a,b).

### 3.3. Effects of Scaffold Components and EGF on Cell Growth

**Figure 4:**
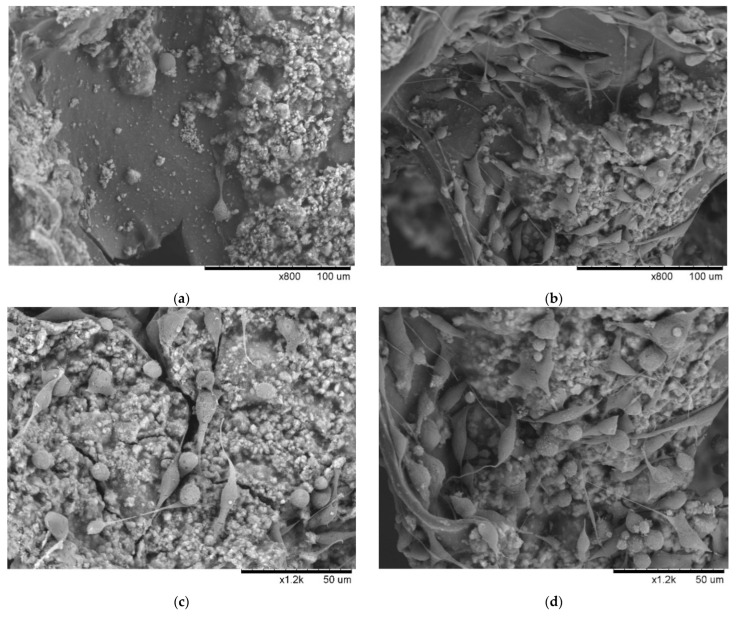
HAp and EGF promoted the adhesion and proliferation of MDA-MB-231: (**a**) on the third day of culture, only a small number of cells were observed to adhere to the smooth surface (gelatin) without EGF; (**b**) cell proliferation for EGF immobilized on the smooth surface (gelatin); (**c**) adhesion of many cells was observed on the rough surface (HAp) under the same conditions; (**d**) mass proliferation of adhered cells when EGF was immobilized on the rough surface (HAp). (Scale bar: 50 µm) (The “×” in scale bar in the figure represents magnification).

The smooth surface in the GHG scaffold consisted of gelatin formed into flakes by crosslinking, and provided a smooth and elastic tissue structure similar to that of the living body. It can be observed from Figure 4a that there were a small number of cells adhering to the position of the smooth tissue; more cell traces were found on the smooth surface at the edge of the rough particles. The rough surface was composed of stacked HAp particles, providing a hard and irregular growth environment different from the smooth, elastic configuration, resulting in calcium similar to the crystallized calcium on the bone tissue in the living body. After three days of culture, it can be seen in Figure 4c that many cells adhered to the rough surface and gaps of the HAp particles, and spread their pseudopodia to expand outward. Additionally, cell-to-cell behavior was also observed on the basis of the pseudopodia. In some cases, some new cells that were spherical in shape were also found around the flattened star-shaped cell population.

The binding between EDC/NHS-streptavidin was a strong covalent bond, meaning that it is difficult for it to fall off [34]. After binding to biotin-EGF and subsequent culture, the non-specifically bound streptavidin lost its activity rapidly during cell culture. Therefore, non-specific binding had little effect on the cells. In addition, streptavidin is a tetramer protein with a very strong affinity for biotin (K_D_ = 10^−1^^5^ M) [35]. The concentration of immobilized streptavidin was in the μg/mL range, while the biotin-EGF used was in the ng/mL regime. Therefore, the streptavidin immobilized on the scaffold was sufficient for biotin-EGF binding.

The EGF (Abcam, Cambridge, UK) standards were prepared, and their absorbance was measured as *C*^1^, while the absorbance of the remaining EGF solution reacted with the scaffold was (*C*^2^). Due to the possibility of EGF inactivation during incubation at low temperature (4 °C), the prepared solution was placed overnight before detection (*C*^0^). The absorbance immobilized on the scaffold was calculated on the basis of the derivation formula:

(1) $$y=C^{1}-C^{2}-\left(C^{1}-C^{0}\right)$$

Then, the value of y calculated was brought into the formula for the standard curve, y=ax+b, to calculate *x*, where *x* the EGF concentration immobilized on the scaffolds. Then, *x* was converted into net weight and brought into the derivation formula to calculate the EGF immobilization rate on the scaffolds:

(2) $$\mathrm{Immobilization}\;\mathrm{rate}=\frac{\mathrm{EGF}\;\mathrm{net}\;\mathrm{weight}\;\mathrm{immobilized}\;\mathrm{on}\;\mathrm{scaffold}}{\mathrm{Net}\;\mathrm{weight}\;\mathrm{of}\;\mathrm{initial}\;\mathrm{prepared}\;\mathrm{EGF}\;\mathrm{solution}}\%$$

The scaffolds in which different concentrations were immobilized were soaked in 10 mL of PBS solution at 37 °C, before taking out and weighing the scaffolds to end incubation. The EGF in the remaining solution was measured and converted into net weight and imported into the formula to calculate the release rate:

(3) $$\mathrm{Release}\;\mathrm{rate}=\frac{\mathrm{Net}\;\mathrm{weight}\;\mathrm{of}\;\mathrm{EGF}\;\mathrm{released}\;\mathrm{in}\;\mathrm{solution}}{\mathrm{EGF}\;\mathrm{net}\;\mathrm{weight}\;\mathrm{immobilized}\;\mathrm{on}\;\mathrm{scaffold}}\%$$

According to the above formula, the immobilization rate of bio-EGF was about 5.6% at an initial concentration of 6.61 nM.

When EGF was immobilized on gelatin in the scaffold, a significant increase in cell number was observed, as well as the coexistence of uniform shape and size, which manifested in various appearances, with spherical, multifilamentary stellate, spindle-shaped, and flat stellate adhesion being observed (Figure 4b). Most cells had filopodia with an average extension length of 35 µm, which is longer than the group without EGF (Figure S3). In addition, it was found that cells with different shapes came into contact with each other by means of pseudopodia, and the pseudopodia were used to adhere to the scaffold surface at different levels in order to support the stranded cells present in the hollow part of the scaffold, which were suspended in appearance. If EGF was built on the surface of HAp, in addition to the observed cells increasing by an order of magnitude, their pseudopodia also showed a slender, filamentous, round, and plump shape, as shown in Figure 4d. Furthermore, the EGF served as an adjunct factor, where it was seen that the cells communicated within a small area via pseudopodia, a large number of cells overlapped with each other, and communication also occurred via reticulopodia. The length of the pseudopodia was also longer (Figure S3).

### 3.4. Effects of EGF on the Proliferation of MDA-MB-231 in Dynamic Culture Based on Composite Matrix

**Figure 5:**
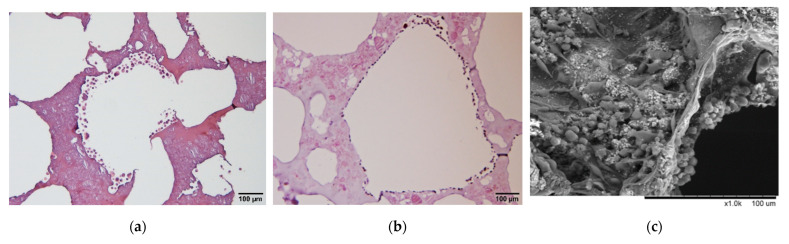

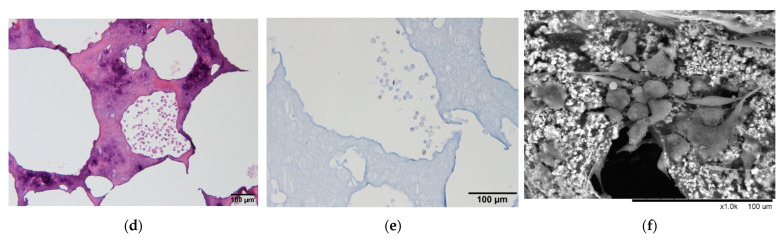
Proliferation of MDA-MB-231 dynamic culture on the seventh day. The (**a**) HE, (**b**) Ki-67, and (**c**) SEM were used, showing the cell proliferative state on the GHG scaffold; if EGF was immobilized on the scaffold massive cell proliferation was revealed, as presented by (**d**) HE, (**e**) Ki-67, and (**f**) SEM. The results indicate that EGF immobilized on the scaffold contributed to the proliferation of MDA-MB-231. (Scale bar: 100 μm) (The “×” in scale bar in the figure represents magnification).

When the cells were dynamically cultured until the 7th day, cell behaviors such as single layers adhering to the scaffold surface transformed into multiple layers and grew, with multilayer growth being observed on the basis of HE staining, but the aggregate sizes were limited by some factors, such as growth range and area (Figure 5a). A biomarker to identify whether it is in an active proliferative state, Ki-67, showed a small amount of Ki-67 protein expression (Figure 5b). Microenvironmental analysis showed a variety of different cellular growth morphologies, such as elongated filopodia or spherical morphologies, in which cells were found growing in a stacked arrangement as spheroids in the corner regions of the scaffold (Figure 5c). In contrast, on an immobilized EGF scaffold, the cells exhibited multilayer growth and cluster formation. It was also observed that cell clusters were induced to aggregate in smaller pores (Figure 5d), with a range of more than 150 μm. The cell numbers expressing −67 protein were also significantly increased, indicating that the EGF contributed to proliferation in the dynamic scaffold culture (Figure 5e). By means of SEM, the tumor-like clusters could be seen in the corners of the holes in which the surrounding cells were clustered. The cells were spherical in shape (Figure 5f). Compared with the non-immobilized EGF group, the growth morphology or behavior was obviously completely different.

Rijal’s team and Ivers’ team pointed out that as cells gained a strong drive, the morphology changed from a flat-shaped epithelial into a round-shaped one that included mesenchymal characteristics [36,37]. It was speculated, on the basis of Figure 5, that once MDA-MB-231 was stimulated by EGF, the stemness was enhanced, and, thus, high motility and transformation into cancer stem cells were both observed [36].

### 3.5. Contribution of EGF to the Behavior of MDA-MB-231 on Scaffold Based on a Multi-Component Matrix

**Figure 6:**
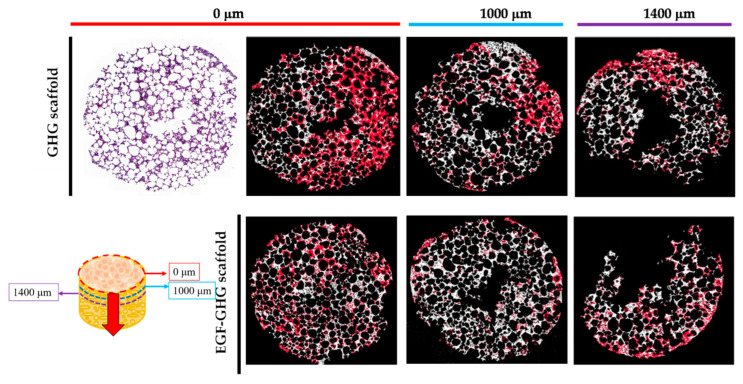
The migration of MDA-MB-231 in dynamic culture until the third day: the scaffold was sectioned from top to bottom (0th, 1000 and 1400 µm) after three days of culture and the effects of EGF on metastasis were observed. The estimated distribution of cells on the GHG scaffold was (from top to bottom) 100, 51, and 31%; when EGF was immobilized on the scaffold, the distribution was 100, 74, and 65%. The cell numbers at the beginning (0th mm) of scaffold slices was set to 100% (scale bar: 2000 µm), respectively.

Since the contrast between cells and scaffolds was too low, it was difficult to directly count the numbers of cells on the scaffold by means of HE staining. Therefore, the obtained stained results were imported into Image J software for image analysis: as the image files were being imported, the Binary program was used to perform image binarization. Later, the cells were counted using the Cell counter in the Plugins program. The red image part shown in Figure 6 was marked cell location.

After three days of dynamic culture, MDA-MB-231 had adhered to the surface of the scaffold, and most of the cell behaviors were at the seeding place (0 µm), so the calculated cell numbers were set to 100%. Subsequent section analysis (500 and 1000 µm) indicated that less cellular behavior was observed in the deeper layers of the scaffold (51% and 31%, respectively). Once the EGF was immobilized on the surface of the scaffold, as a result of the matrix making up the scaffold providing cells with nutrition and adhesion, the EGF supplied the ability to promote cell behavior, including proliferation and migration. Therefore, the distribution of cells was not limited to the locations of the initial seeding, and more cellular behavior was observed in the deeper scaffolds (100%, 74%, and 65%, respectively, as shown in Figure 6). In addition, the function of EGF increased the number of cells by nearly 10% at the third slice (1400 µm), more than doubling in proportion. It was shown that EGF immobilized on the scaffold matrix did indeed contribute to cellular proliferation and migration. Our experimental results are consistent with the metastases of breast cancer bone described in Dr. Thibaudeau’s article [15].

## 4. Discussion

The main purpose of this study was to investigate the dynamic progression of triple-negative breast cancer in bone metastases by multi-component bone matrix. MDA-MB-231 presented abundant EGFR expression on the cell membrane surface [38,39], which served as a template for exploring the development of cell behavior as a dynamic progression. Gelatin and HAp were used as the main matrix for synthesizing scaffold via cross-linking, with the aeration during preparation making its porous structure similar to that of the human trabecular bone [30]. Of these two materials, HAp is a simulated bone component (calcium phosphate, calcium carbonate, calcium fluoride, etc.), while gelatin is similar to the colloidal filler in bone pores. Analysis using the OCPC method showed that a small amount of calcium ions was released from this scaffold on the third and seventh days. The properties of the calcium ions released from scaffold were helpful for the growth of cells adhering to the scaffold [40,41].

The three main matrices making up the bone-like scaffold had important and distinct missions at each stage of the tumor cell culture. Gelatin provided the necessary nutrients for cell adhesion; Hap, which was constructed on the gelatin, provided a favorable environment for cell adhesion; and EGF enhanced cell proliferation and migration ability with respect to gelatin and HAp. In this context, Figure 5 shows that the MDA-MB-231 demonstrated obvious proliferation on the above-mentioned multi-component material (EGF-HAp-gelatin), and at the same time, it was transformed into a fusiform shape with the potential for metastasis (Figure 4d). EGF immobilized on the scaffold matrix does indeed contribute to cellular proliferation, and deep distributions implying specific metastases are also validated in Figure 6. The same trend was also revealed in the increased length of the pseudopodia (Figure S3).

**Figure 7:**
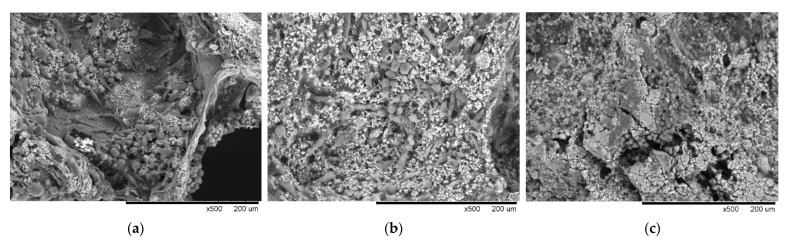
MDA-MB-231 metastasis on GHG scaffold: (**a**) MDA-MB-231 was dynamically cultured in GHG scaffold until the seventh day, and it was observed that the cells proliferated/metastasized slowly toward two-dimensional planes on the scaffold; (**b**) the EGF was immobilized on the scaffold where cells proliferated/metastasized in the direction of the hole in a three-dimensional manner; (**c**) MCF-7 was dynamically cultured under the conditions of (**a**) until the seventh day, at which point a similar phenomenon to that described in (**b**) could also be observed. (Scale bar: 200 µm) (The “×” in scale bar in the figure represents magnification).

Dr. Wang’s and Dr. Liverani’s teams presented incisive insights on hypoxia-driven cancer progression, respectively [1,42]. When MDA-MB-231 was cultured on our dynamic EGF-GHG scaffold until the seventh day, the cells demonstrated enhanced cell–cell interaction (contact) and massive proliferation towards the holes of the scaffold, which was corroborated by the high expression of HIF-1α of MCF-7 in the GHG scaffold cultured under the same conditions exhibiting similar results (Figure 7). This indicated that our EGF-GHG scaffold created a similar hypoxia-driven cancer culture progression model under the dynamic culture state of normal oxygen supply.

**Figure 8:**
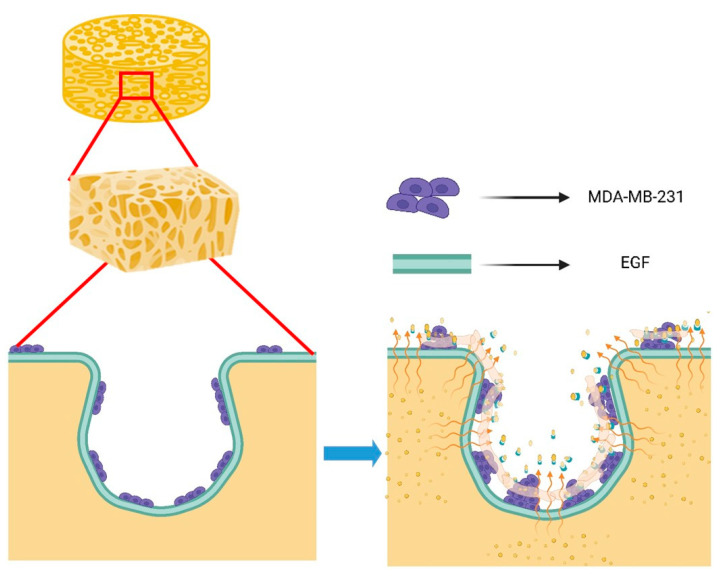
Schematic diagram of dynamic scaffolding three-dimensional hypothesis: While MDA-MB-231 adhered to the scaffold, its proliferation was flattened; the EGF was immobilized, and thus, the scaffold degraded into small fragments that were distributed around the pores inside the scaffold, and the MDA-MB-231-MDA-MB-231 interaction between the scaffold and the fragments was directed towards spheroids. (This Figure was created with BioRender.com (accessed on 12 August 2022)).

In this article, we described an in vitro microenvironment simulating cancer cells in bone with respect to adhesion, proliferation and three-dimensional growth, as shown in Figure 8. On the basis of the multi-component matrix constituting a bone-like scaffold providing the needs of cells in different growth stages, degradable gelatin promoted cancer cell growth on the scaffold surface toward 3D aggregation, and EGF constructed on the scaffold matrix sped up cancer cell adhesion, proliferation and migration. We assume that cell–cell interactions were triggered, thereby progressing three-dimensional cell growth (spheroid) (Figure 5f). The developed dynamic culture system can be used to study the mechanism of dynamic local tumor microenvironment changes [43] and the study of tissue types [44].

## 5. Conclusions

We constructed a dynamic scaffold for the culture of tumor spheroids that had metastasized in situ to bone tissue, providing functions such as nutrition, cell adhesion, and proliferation/metastasis at different stages, and guiding progression in three dimensions. This dynamic scaffold has great potential for culturing the tissue-like or organoid properties of two different structures, thereby simulating the characteristic state of actual in vivo tissues or organs. This dynamic scaffold culture module could provide indicative and constructive suggestions at the tissue level for drug screening in the future.

## Figures and Tables

**Table 1:** Comparison of calcium ion release and EGF concentration in the GHG scaffold with the breast cancer cells numbers ^1,2^.

		GHG Scaffold	
Time (Day)	Ca^2+^ Conc. (mM)	Absent EGF	6.61 EGF (nM)
0	0	100.0 ± 13.01	
3	0.0824 ± 0.0064	119.2 ± 3.82	150.0 ± 14.14
7	0.1126 ± 0.0002	147.2 ± 16.12	170.0 ± 4.60

^1^ Cell number: ×10^4^. ^2^ MDA-MB-231.

## Data Availability

All the data relevant to the study are included in the article.

## Supplementary Materials

The following supporting information can be downloaded at: https://www.mdpi.com/article/10.3390/polym14163340/s1, Figure S1. Bioreactor: (a) schematic diagram of the bioreactor; the holes around the syringe and the rotation of the stirrer below drive the disturbance of the culture medium, thereby achieving the effect of internal and external flow; (b) the composition of the bioreactor: 20 mL transparent glass jar, rubber stopper, cut and perforated syringe and stirrer. (i: transparent jar; ii: rubber bung; iii: cropped syringe; iv: stirrer); Figure S2. Simple flow chart of dynamic culture; Figure S3. Pseudopodia measurement of MDA-MB-231 in dynamic culture on scaffold: the cell pseudopodia length adhered and proliferated on scaffold was measured for 3 days of dynamic culture. It was found that the cell pseudopodia in the gelatin part were shorter than those in the HAp part (20 vs. 25 µm); when EGF was used, a reduction in the difference in pseudopodia length was observed between the two materials (35 vs. 38 µm).
