# Supplementary material for: A Tumor Accelerator Based on Multicomponent Bone Scaffolds and Cancer Cell Homing
Source: Polymers (Basel). 2022 Aug 16;14(16):3340. doi: 10.3390/polym14163340 (PMC9416103; doi:10.3390/polym14163340)
Supplement: Supplementary file 1 [file polymers-14-03340-s001.zip › polymers-1870100-supplementary.pdf]

polymers

Article

# A Tumor Accelerator Based on Multicomponent Bone Scaffolds and Cancer Cell Homing

**Chen-Ji Huang [1], Pei-Kuan Chou [1,2], Zong-Yi Sher [1,2], You-Rong Chen [1,3], Tan-Yueh Chen [1] and Guo-Chung Dong [1,2,3,*]**

[1] Institute of Biomedical Engineering and Nanomedicine, National Health Research Institutes, Miaoli 35053, Taiwan
[2] Graduate Program of Biotechnology in Medicine, National Tsing Hua University, Hsinchu 300044, Taiwan
[3] International Master Program of Translational Medicine, National United University, Miaoli 360301, Taiwan
* Correspondence: gcdong@nhri.org.tw; Tel.: +886-37-246166#37135

**Supplementary Materials:**

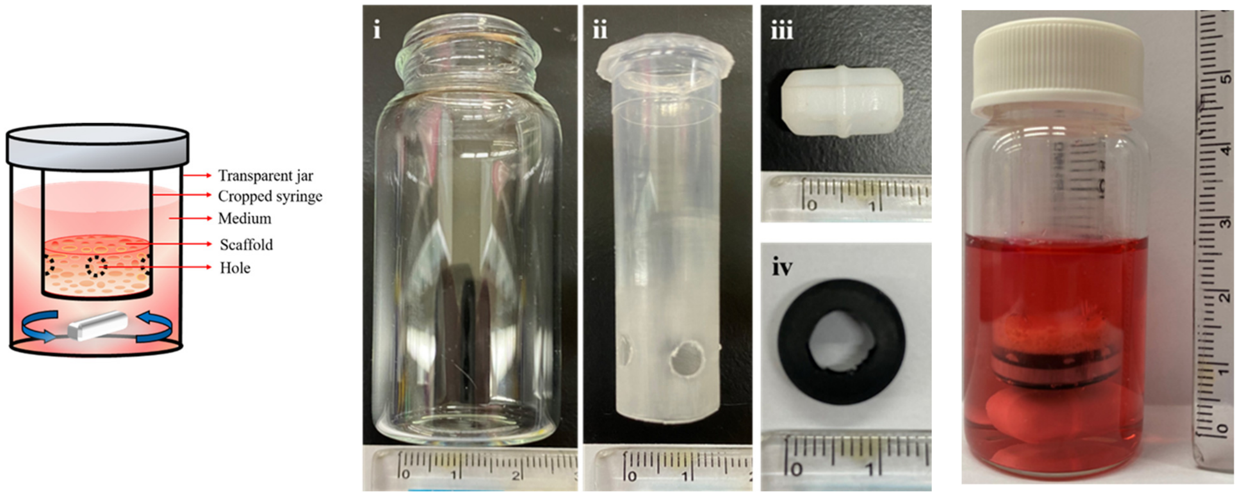


(**a**) (**b**) (**c**)

**Figure S1.** Bioreactor: (**a**) schematic diagram of the bioreactor; the holes around the syringe and the rotation of the stirrer below drive the disturbance of the culture medium, thereby achieving the effect of internal and external flow; (**b**) the composition of the bioreactor: 20 mL transparent glass jar, rubber stopper, cut and perforated syringe and stirrer (i: transparent jar; ii: rubber bung; iii: cropped syringe; iv: stirrer). (**c**) Entity diagram of the dynamic culture of bioreactor.

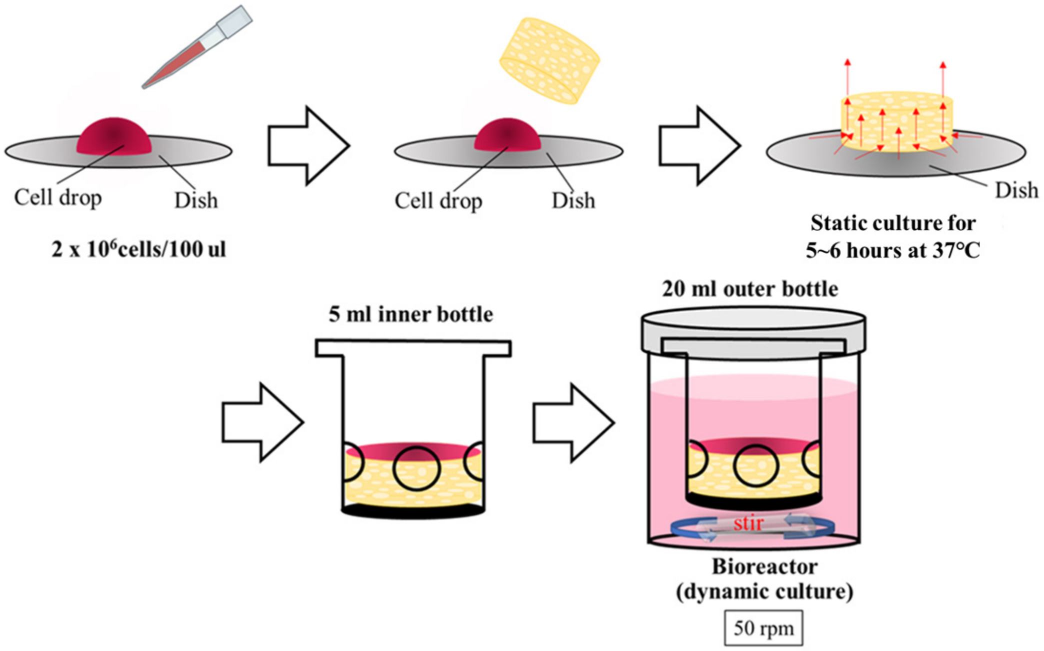


**Figure S2.** Simple flow chart of dynamic culture.

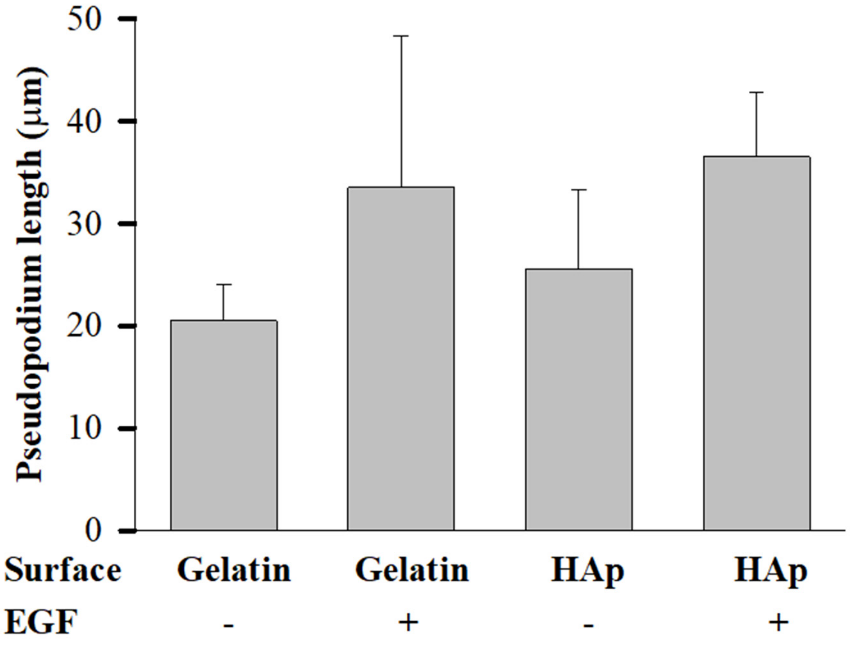


**Figure S3.** Pseudopodia measurement of MDA-MB-231 in dynamic culture on scaffold: the cell pseudopodia length adhered and proliferated on scaffold was measured for 3 days of dynamic culture. It was found that the cell pseudopodia in the gelatin part were shorter than those in the HAp part (20 vs. 25 μm); when EGF was used, a reduction in the difference in pseudopodia length was observed between the two materials (35 vs. 38 μm).
